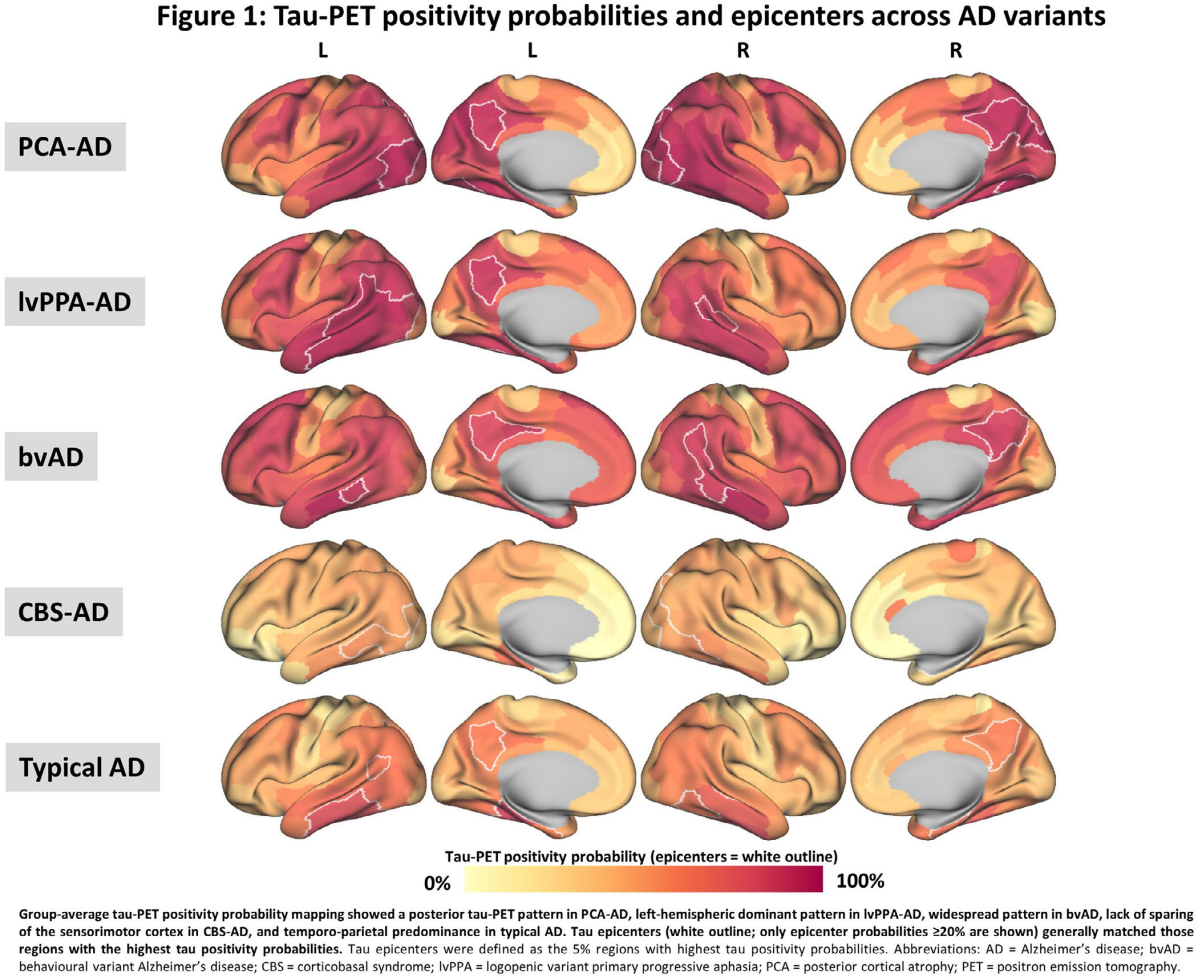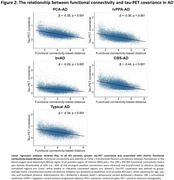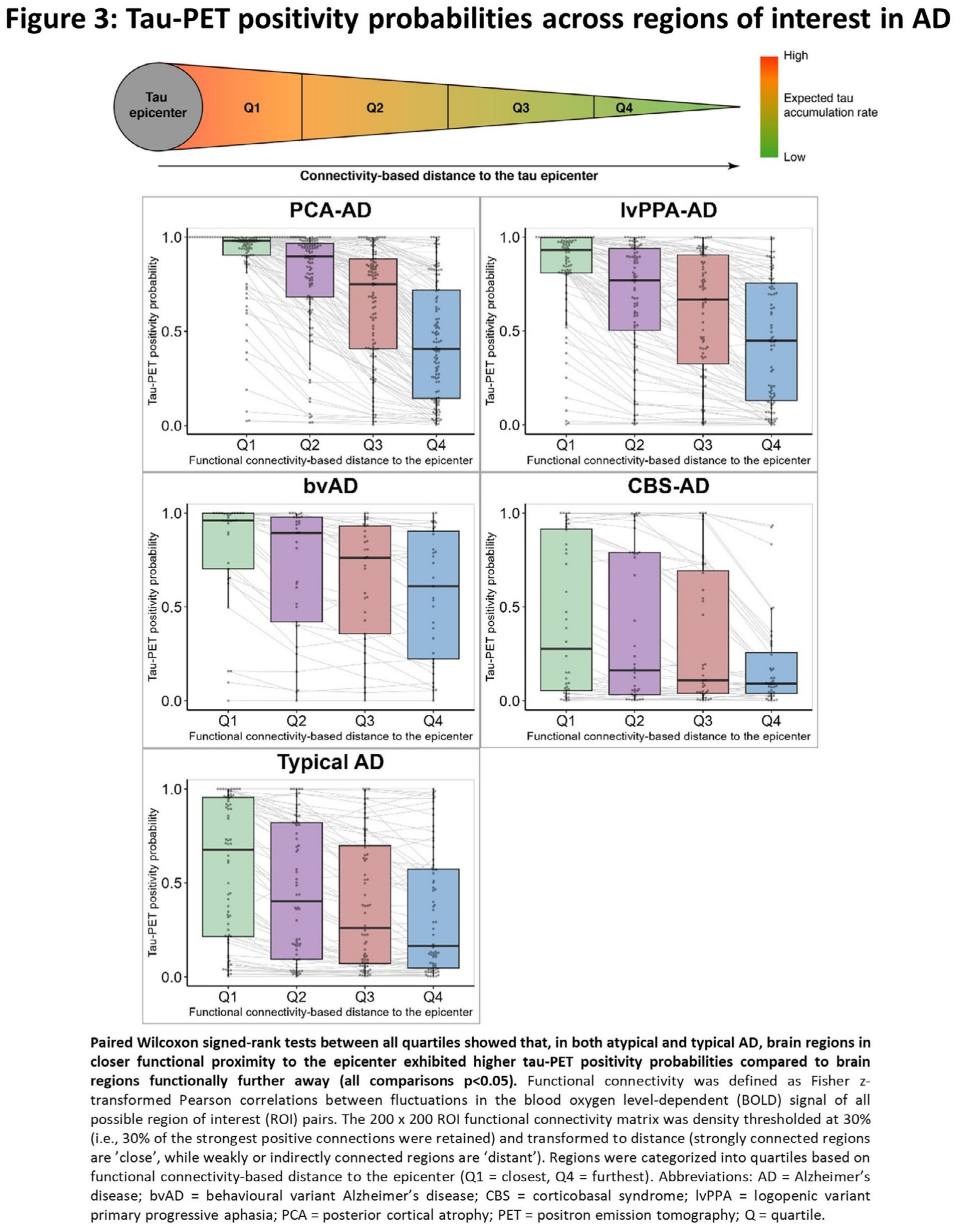# Connectivity as a universal predictor of tau spreading in typical and atypical Alzheimer’s disease

**DOI:** 10.1002/alz.085869

**Published:** 2025-01-09

**Authors:** Hannah de Bruin, Colin Groot, ADNI, Henryk Barthel, Gérard N Bischof, Ronald Boellaard, Matthias Brendel, David M Cash, William Coath, Gregory S Day, Brad C Dickerson, Elena Doering, Alexander Drzezga, Christopher H. van Dyck, Thilo van Eimeren, Wiesje M. van der Flier, Carolyn Fredericks, Tim D Fryer, Elsmarieke van de Giessen, Brian A. Gordon, Jonathan Graff‐Radford, Diana A Hobbs, Günter Höglinger, Merle C Hönig, David J Irwin, P Simon Jones, Keith A. Josephs, Yuta Katsumi, Eddie B Lee, Johannes Levin, Maura Malpetti, Scott M McGinnis, Adam P Mecca, Ilya M. Nasrallah, John T O'Brien, Ryan S O'Dell, Carla Palleis, Robert Perneczky, Jeffrey S Phillips, Yolande A.L. Pijnenburg, Deepti Putcha, Nesrine Rahmouni, Pedro Rosa‐Neto, James B Rowe, Michael Rullmann, Osama Sabri, Dorothee Saur, Andreas Schildan, Jonathan M Schott, Matthias L Schroeter, Stijn Servaes, Irene Sintini, Jenna Stevenson, Joseph Therriault, Alexandra Touroutoglou, Anne E Trainer, Denise Visser, Philip SJ Weston, Jennifer L. Whitwell, David A Wolk, Nicolai Franzmeier, Rik Ossenkoppele

**Affiliations:** ^1^ Alzheimer Center Amsterdam, Neurology, Vrije Universiteit Amsterdam, Amsterdam UMC location VUmc, Amsterdam Netherlands; ^2^ Amsterdam Neuroscience, Neurodegeneration, Amsterdam Netherlands; ^3^ Institute for Stroke and Dementia Research, Klinikum der Ludwig‐Maximilians Universität München, Munich Germany; ^4^ Lund University, Clinical Memory Research Unit, Lund Sweden; ^5^ Alzheimer’s Disease Neuroimaging Initiative, http://adni.loni.usc.edu/, CA USA; ^6^ Department of Nuclear Medicine, University of Leipzig, Leipzig Germany; ^7^ Research Center Jülich, Institute for Neuroscience and Medicine II, Molecular Organization of the Brain, Jülich Germany; ^8^ University of Cologne, Faculty of Medicine and University Hospital Cologne, Department of Nuclear Medicine, Cologne Germany; ^9^ Radiology & Nuclear Medicine, Vrije Universiteit Amsterdam, Amsterdam UMC location VUmc, Amsterdam Netherlands; ^10^ Amsterdam Neuroscience, Brain Imaging, Amsterdam Netherlands; ^11^ Department of Nuclear Medicine, University Hospital, LMU Klinikum, Munich, Bavaria Germany; ^12^ Dementia Research Centre, UCL Queen Square Institute of Neurology, London UK; ^13^ Dementia Research Centre, UCL Queen Square Institute of Neurology, University College London, London UK; ^14^ Mayo Clinic, Jacksonville, FL USA; ^15^ Frontotemporal Disorders Unit, Department of Neurology, Massachusetts General Hospital and Harvard Medical School, Boston, MA USA; ^16^ German Center for Neurodegenerative Diseases (DZNE), Bonn/Cologne Germany; ^17^ Alzheimer's Disease Research Unit, Yale School of Medicine, New Haven, CT USA; ^18^ University of Cologne, Faculty of Medicine and University Hospital Cologne, Department of Neurology, Cologne Germany; ^19^ Yale University School of Medicine, New Haven, CT USA; ^20^ Department of Neurology, Yale‐New Haven Hospital, New Haven, CT USA; ^21^ Department of Clinical Neurosciences and Cambridge University Hospitals NHS Trust, University of Cambridge, Cambridge UK; ^22^ Wolfson Brain Imaging Centre, University of Cambridge, Cambridge UK; ^23^ Department of Radiology, Washington University in St. Louis, St. Louis, MO USA; ^24^ Knight Alzheimer's Disease Research Center, Washington University in St. Louis, St. Louis, MO USA; ^25^ Department of Neurology, Mayo Clinic, Rochester, MN USA; ^26^ Department of Neurology, Klinikum der Ludwig‐Maximilians Universität München, Munich Germany; ^27^ Department of Neurology, University of Pennsylvania, Philadelphia, PA USA; ^28^ Penn Frontotemporal Degeneration Center, University of Pennsylvania, Philadelphia, PA USA; ^29^ Institute on Aging, University of Pennsylvania, Philadelphia, PA USA; ^30^ Department of Pathology & Laboratory Medicine, University of Pennsylvania, Philadelphia, PA USA; ^31^ Center for Neurodegenerative Disease Research, University of Pennsylvania, Philadelphia, PA USA; ^32^ Department of Neurology, LMU University Hospital, LMU Munich, Munich Germany; ^33^ Munich Cluster for Systems Neurology (SyNergy), Munich Germany; ^34^ German Center for Neurodegenerative Diseases (DZNE), Munich Germany; ^35^ Frontotemporal Disorders Unit and Massachusetts Alzheimer's Disease Research Center, Department of Neurology, Massachusetts General Hospital and Harvard Medical School, Boston, MA USA; ^36^ Alzheimer's Disease Research Unit, Yale University School of Medicine, Department of Psychiatry, New Haven, CT USA; ^37^ Department of Radiology, University of Pennsylvania, Philadelphia, PA USA; ^38^ Department of Psychiatry, University of Cambridge, Cambridge UK; ^39^ Department of Psychiatry and Psychotherapy, Klinikum der Ludwig‐Maximilians Universität München, Munich Germany; ^40^ Department of Neurology, Perelman School of Medicine, University of Pennsylvania, Philadelphia, PA USA; ^41^ Alzheimer Center Amsterdam, Neurology, Vrije Universiteit Amsterdam, Amsterdam UMC, Amsterdam Netherlands; ^42^ McGill University Research Centre for Studies in Aging, Montreal, QC Canada; ^43^ Translational Neuroimaging Laboratory, The McGill University Research Centre for Studies in Aging, Montréal, QC Canada; ^44^ Medical Research Council Cognition and Brain Sciences Unit, Cambridge UK; ^45^ Department of Neurology, University of Leipzig, Leipzig Germany; ^46^ Clinic for Cognitive Neurology, University of Leipzig, Leipzig Germany; ^47^ Max Planck Institute for Human Cognitive and Brain Sciences, Leipzig Germany; ^48^ McGill Centre for Studies in Aging, Department of Neurology and Neurosurgery, McGill University, Montreal, QC Canada; ^49^ Department of Radiology, Mayo Clinic, Rochester, MN USA; ^50^ Clinical Neurosciences Imaging Center, Yale University School of Medicine, New Haven, CT USA; ^51^ UK Dementia Research Institute, London UK; ^52^ Penn Memory Center, University of Pennsylvania, Philadelphia, PA USA; ^53^ University of Gothenburg, The Sahlgrenska Academy, Institute of Neuroscience and Physiology, Psychiatry and Neurochemistry, Gothenburg Sweden; ^54^ Munich Cluster for Systems Neurology (SyNergy), Munich, Bavaria Germany

## Abstract

**Background:**

There is a strong link between tau and progression of Alzheimer’s disease (AD), necessitating an understanding of tau spreading mechanisms. Prior research, predominantly in typical AD, suggested that tau propagates from epicenters (regions with earliest tau) to functionally connected regions. However, given the constrained spatial heterogeneity of tau in typical AD, validating this connectivity‐based tau spreading model in AD variants with distinct tau deposition patterns is crucial.

**Method:**

We included 269 amyloid‐β‐positive (PET/CSF) individuals with clinically diagnosed atypical AD (113 posterior cortical atrophy, PCA‐AD; 83 logopenic variant primary progressive aphasia, lvPPA‐AD; 33 behavioural variant AD, bvAD; 40 corticobasal syndrome, CBS‐AD) and 68 with typical AD from 12 international cohorts, who underwent tau‐PET (54% [^18^F]AV1451/[^18^F]flortaucipir/Tauvid, 27% [^18^F]MK6240, 19% [^18^F]PI2620). Using Gaussian mixture modeling including amyloid‐β‐negative controls, cross‐sectional tau‐PET standardized uptake value ratios within Schaefer‐200 atlas regions were transformed to tau positivity probabilities. Tau epicenters were defined as the 5% regions with highest tau positivity probabilities. For each variant, the association between functional connectivity‐based distance (using the 30% strongest positive region‐to‐region connections of a group‐average connectivity matrix from ADNI elderly controls) and tau‐PET covariance (group‐average correlation per region pair) was assessed through linear regression, adjusting for age, sex, site, and Euclidean distance. Regions were categorized based on functional proximity to the epicenter (quartiles 1‐4) and tau positivity probabilities were assessed accordingly.

**Result:**

Tau positivity probabilities matched clinical variants, with a posterior pattern in PCA‐AD, left‐hemispheric dominant pattern in lvPPA‐AD, widespread pattern in bvAD, sensorimotor cortex involvement in CBS‐AD, and temporo‐parietal predominance in typical AD (Figure 1). In line with this, tau epicenters were highly heterogeneous across variants (Figure 1). In all variants, greater tau‐PET covariance was associated with shorter functional connectivity‐based distance (Figure 2). We observed that regions in closer functional proximity to the epicenter exhibited higher tau positivity probabilities than regions functionally further away (p<0.05, Figure 3).

**Conclusion:**

This multi‐center study shows that the brain’s functional architecture serves as a universal predictor of tau spreading in AD. Since tau is a key driver of neurodegeneration and cognitive decline in AD, this finding holds potential for personalized medicine and defining participant‐specific endpoints in clinical trials.